# The application of certified reference materials for clinical mass spectrometry

**DOI:** 10.1002/jcla.24301

**Published:** 2022-03-11

**Authors:** Shen Lingxiao, Ma Jinfei, Yang Qingqing, Liu Pengyun, Liu Huafen

**Affiliations:** ^1^ Hangzhou Calibra Diagnostics Co., LTD Hangzhou China

**Keywords:** application, calibration solution, clinical mass spectrometry, laboratory, reference material

## Abstract

**Background:**

In the application process of clinical mass spectrometry, there are difficulties such as standardization, low degree of automation, complex instruments, and high requirements for operations, which will affect the accuracy and comparability of results. Reference materials are one of the major approaches to achieve measurement accuracy and metrological comparability.

**Methods:**

Based on the problems of reference materials in clinical mass spectrometry, the precautions for the use of reference materials are summarized in the aspects of measurement method validation, calibrator usage, and quality control in this article. Additionally, combined with the previous experience of the author's laboratory, the operation mode and acceptance criteria of the new calibration solution lot replacement were formulated to ensure the continuous comparability of the measurement system.

**Conclusion:**

Carefully understand and correctly regulate the use and management of reference materials to ensure the accuracy of test results, thereby improving the comparability and consistency of results between laboratories.

## Graphical abstract

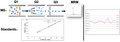

## 1 THE IMPORTANCE OF REFERENCE MATERIALS IN CLINICAL MASS SPECTROMETRY

Mass spectrometry technology has been continuously used in clinical testing by its high specificity, high sensitivity, and simultaneous analysis of multiple compounds,^1^ but the application process has difficulty in standardization, low automation, complex instruments, high expectation of the laboratory technicians, etc..^2^ These deficiencies will affect the accuracy and comparability of the clinical test results. These results have composed over 80% of the auxiliary information for clinical diagnoses and treatments,^3^ which shows the importance of the accuracy and comparability of test results.^4^ Therefore, the application of clinical mass spectrometry also requires the joint efforts of all parties. One of the ways that can be adopted is to use reference methods and reference materials to establish and ensure the traceability of the test results^5^, ^6^; and the reference materials, used as one of the key elements of traceability system of clinical tests, ensure the comparability and accuracy of test results in time and space. However, how to select suitable reference materials and reasonably apply them to clinical mass spectrometry technology is still a problem.

## 2 THE PROBLEMS OF REFERENCE MATERIALS APPLICATION IN CLINICAL MASS SPECTROMETRY

### 2.1 The main problem when having a complete traceability chain

Due to the difficulty of obtaining human samples and the timeliness of diagnosis, clinical test aims to develop and apply on small samples, easy automation,^3^, ^7^ as well as focus on the application of supporting commercial reagents, such as new screenings and 25‐hydroxyvitamin D_3_ in mass spectrometry technology.

At the 2020 Clinical Mass Spectrometry Application Conference and Mass Spectrometry Reagent Exhibition, experts have pointed out that the 25‐hydroxyvitamin D_3_ screening in laboratory developed test (LDT) and the average coefficient of variation (CV%) of commercial reagents is 9.3% and 9.5%, respectively; the average CV% of LDT and commercial reagents of 25‐hydroxyvitamin D_2_ are 11.5% and 12.2%, respectively. It shows that the quality of current commercial reagents does not have substantial advantage compared with LDT. The reason could be that the calibrator indicators provided by the reagent manufacturers, such as the accuracy of 80%–120% and differences between lots of no more than 20%, are too wide to accept the standard, the preparation method of the calibration solution is inconsistent, and the IVD manufacturers’ traceable certified reference materials or traceability are inconsistent, etc.^8^, ^9^

### 2.2 The main problem when missing a complete traceability chain

Commercial reagents in clinical mass spectrometry technology have certain limitations in practical applications.^1^ For example, CFDA shows that as of March 2020, only five types of kit products contain calibrators (Table 1). Obviously, with the increasing demand for personalized medicine and precision medicine, these kits cannot meet the testing needs. Therefore, clinical mass spectrometry laboratories often build their own detection methods based on the flexibility of mass spectrometry.

**TABLE 1:** Summary of CFDA‐approved clinical mass spectrometry kit information

Program	Import	Class	Number of valid kit registrations	Basic ingredients
Amino acid and carnitine check	No	III	4	Calibrator, mobile phase, internal standard, quality control, extraction solution, consumables, etc.
	Yes	III	4	Calibrator, mobile phase, internal standard, quality control, extraction solution, consumables, etc.
25‐Hydroxy vitamin D	No	II	6	Calibrator, mobile phase, internal standard, quality control, extraction solution, etc.
Homocysteine	No	II	2	Calibrator, mobile phase, internal standard, quality control, extraction solution, etc.
Glycocholic acid	No	II	1	Mobile phase, internal standard, quality control, extraction solution, etc.
1,5‐Anhydroglucitol	No	II	2	Calibrator, internal standard, quality control, extraction solution, etc.
Cortisol	No	II	1	Calibrator, internal standard, quality control, etc.

Note

Information was collected till April 2020.

The results of the 2019 aldosterone accuracy verification survey organized by the Chinese National Center for Clinical Laboratories showed that the CV%s of different concentrations of aldosterone among 20 clinical mass spectrometry laboratories are: 29.8% ‐ 51.3%. The deviation of test results between two of the largest laboratories can reach as high as 729%,^10^ indicating huge differences between test results from different clinical mass spectrometry laboratories. The influencing factors of mass spectrometry technology include pre‐processing methods, chromatographic columns, instruments, calibrators, internal standards, and personnel proficiency, etc., which cause errors or deviations in the results. Currently, “Recommendations for the Development and Management of Self‐built Testing Methods in my country's Medical Testing Departments”,^11^ “Recommendations for the Clinical Application of Liquid Chromatography‐Mass Spectrometry”,^12^ and “Development and Verification of Liquid Chromatography Tandem Mass Spectrometry Clinical Testing Methods”,^13^ etc., have pointed out method development and verification, quality control, personnel requirements, etc., among these guidelines. Each laboratory has developed their own reference standards to implement these guidelines, but why there still exists such huge differences among the results?

First, since the supply of certified reference materials cannot fully meet the need of all aspect,^14^ this has caused the laboratory’ LDT method to be unable to carry out effective measurement method confirmation; Secondly, differences among different matrix from different calibration solutions, different sources of the calibrators lead to the lack of interoperability. In addition, the inability to obtain suitable quality control materials when carrying out indoor quality control results in the failure of reflecting system deviations during long‐term quality control. The above factors lead to inaccurate results even when other aspects of the measurement method are implemented as required.

## 3 PRECAUTIONS FOR THE APPLICATION OF REFERENCE MATERIALS IN MASS SPECTROMETRY

Reference materials are widely applied in the confirmation, calibration, metrological traceability, quality control, etc., of the measurement methods by their manufacturers. In the field of clinical mass spectrometry, manufacturers and laboratories also involved in the application of reference materials. However, due to the lack of uniform standards, a lot of aspects still need to be paid attention to when in applications.

### 3.1 Application of reference materials in the confirmation of measurement methods in clinical mass spectrometry

The current development of clinical mass spectrometry technology mainly relies on first class kits or LDT methods. Thus, laboratories can use certified reference materials to verify the accuracy of the measurement method confirmation. Laboratories can participate into accuracy verification plan by authoritative organizations. For example, 25‐hydroxy vitamin D_3_ program can participate into the NCCL‐C‐35 vitamin accuracy verification plan by Chinese National Center for Clinical Laboratories; the Accuracy‐Based Vitamin D program (ABVD) by the College of American Pathologists, or the Vitamin D External Quality Assessment Scheme (DEQAS), etc. Additionally, laboratories can also seek other suitable certified reference materials from JCTLM official website to perform accuracy verification on their own and effectively establish measurement results of metrological traceability. When preforming the verification of accuracies, laboratories should try to cover the whole application areas of measurement methods, including same matrix type, same concentration level of the sample; if situation permits, laboratories can perform evaluation on certified reference materials with different concentration levels.

When preforming the verification of the precision of the measurement method confirmation, the laboratories can use reference materials that are sufficiently uniform and stable, even though they do not have definite value or measurement traceability when in use of the verification of precision.

### 3.2 Application of reference materials as calibrators in clinical mass spectrometry

At the beginning of each project, the laboratory combined with the “Clinical Application of Liquid Chromatography‐Mass Spectrometry”^12^ recommends, “when purchasing and using commercialized standard products, and the commercialized reference materials cannot be obtained, laboratories can consider preparing the reference materials themselves, to make sure that the matrix of the calibrator is as similar to the clinical sample as possible.” When the above situation happens, laboratories should choose verified reference materials as priority, if not available, then choose high‐purity materials with uncertainty, as well as choose reliable suppliers. Meanwhile, laboratories should be careful that once the calibrator is in use, the calibrator should not be in use of the accuracy's verification and quality control.

The purchased calibrators often come as powers, commercialized freeze‐dried materials, or high‐concertation solutions, which cannot be used directly, and further preparation of matrix reference materials or reference solutions are required by weighing, mixing, diluting, etc. To ensure reliability and accuracy, our laboratories always make sure of the excellent quality control from purchasing calibrators to the preparation process.

1. Acceptance of the purchased calibrator.
2. Weighing equipment: the calibrator purchased for the laboratories is often in the milligram level, so it is recommended to use a one‐millionth balance for weighting. However, one‐millionth balance is very susceptible to external factors, and many factors need to be considered when it is put into use: (a). The weighing room should be free of vibration and air flow; the weighing platform should be stable, anti‐magnetic, and anti‐static; (b). Keep it away from direct light, try to use fluorescent tubes, avoid close to air‐conditioning vents, ceiling fans, windows or radiators, etc.; also avoid placing it biological safety cabinets, fume hoods, etc. (c). Keep the ambient temperature between 10–30°C, as well as a low temperature fluctuation. The humidity is recommended to be maintained at 45%–60% and 20%–80% as maximum; when humidity is lower than 40%, glasses, plastics, powers, particles, or other low‐conductivity materials cannot easily eliminate static electricity, laboratories should increase the air humidity and use metal containers instead; (d). Ensure that the balance is adequately preheated before use, and the temperature of the weighing sample is consistent with the temperature of the weighing chamber; (e). Strictly follow the operating requirements to operate and verify.
3. Preparation equipment: the equipment used for the preparation of the calibration solution should be cleaned according to the project requirements to ensure that the preparation is free of interference and will not affect the test results.
4. Pipettes, volumetric flasks, etc., used for quantification should be qualified to prevent systemic deviations in the results due to inaccurate quantification during the preparation.
5. When preparing the calibration solutions, just as the manufacturer's system, laboratories must use interchangeable calibrators to ensure that when same patient sample is measured with different manufacturer's measurement systems there will be consistent test results.^8^, ^15^ Laboratories also need to pay attention when selecting same or similar matrix as the actual sample; otherwise, the difference with fresh sample may lack interoperability.^16^ During the addition process, laboratories should be mindful of the volume of added solution to avoid significant changes in the sample matrix. If the matrix of the calibration solution is quite different from the actual sample, laboratories should consider conducting a study of extraction efficiency.
6. After preparation, the calibration curve should be stored and packed in a clean container that meets the requirements, relevant records and labels should be made to ensure that the source of calibration curve lot can be traced.
7. Other precautions: Some of the calibrators may be sold by the companies importing foreign products in sub‐packages. However, due to poor design control during the packing, storage and transportation, lack of design understanding of the imported products, or lack of relevant parameters and technical data, etc., it is easy to cause large differences in calibrators between bottles and between different brands.^17^ Therefore, before the formal preparation of calibration solution, we can consider diluting different brands or different lots of calibrators to the same concentration and perform detection in the form of AB crossover and decide whether the lot of calibrators are applicable according to the difference in the peak area.

When the calibrator is prepared as calibration solution, the inspection laboratory often prepares it in lots due to the stability of the system. At this time, whether the prepared calibration solution is applicable need to be carefully estimated. If the new calibration solution is used as the unknown sample to set the value with the old calibration solution as the conventional method, when the detection value of each concentration of the new calibration solution is compared with the theoretical value, and accept ±15% as acceptable. It is easy to cause overall deviation of the system due to large acceptance criteria. Since the calibration solution is also part of the measurement system, the measurement system changes when the calibration solution changes. Therefore, the author's laboratory has formulated the comparison of operation method and acceptance criteria after the lots change of calibration solution (Table 2).

**TABLE 2:** Similarities and differences between lot replacement of old and new calibration solutions in different standards

	Author's laboratory	C62‐A	Chinese Pharmacopeia: 9012 guidelines for validation of methods for quantitative analysis of biological samples	Recommendations for clinical application of LC‐MS/MS
Methods	New calibrator lots may be validated by analyzing a group of patient samples at a time when the method was calibrated using the old lot of calibrators The same patient samples are then reanalyzed when the method is calibrated using the new lot		No lot change requirements; Only the bracket setting requirements under each lot are specified (at least 6 calibration standard samples)	
	Analyte content ideally covers the entire analytical measurement range, including medically determined levels	Alternatively, the new lot of calibrators may be analyzed as unknown samples when the method is calibrated using the old lot		
Acceptance Criteria	The slope in the linear regression equation of the old and new calibration curves is between 0.9–1.1, and long‐term monitoring of the lot change coefficient to prevent its overall bias. The sample bias at the medical decision point should be ≤1/3 TEa	No acceptance criteria specified	More than 75% of the back‐calculated concentration is in the range of ±20% of the declared value (LLOQ and HLOQ ± 25%)	Concentration deviation at LLMI should be within ±20%, the remaining concentration deviation should be within ±15%

Choose at least 20 pieces of fresh serum from patients with different concentrations. The analyte content should preferably cover the entire analytical measurement range, including the medical decision level. Together with the new and old lot of calibration solutions, the analyte will be processed and tested in accordance with the standard operating procedures. Then, graph the old and new calibration curves separately, and calculate the regression equation *Y* = *a*X + *b* after setting values for the patient sample lots.

1. Firstly, the slope a is recommended to be within the range of 0.9–1.1, which is when the total allowable error of the analyte is 30%, if the slope of the calibration solution meets the requirements when the lot number of the calibration solution is changed, the system offset will be <10%, and the overall detection result will be within 10% in subsequent applications.
2. The critical value of the medical decision level is substituted into the linear regression equation as the X_critical value_, and compared the obtained Y_critical value_ with the X_critical value_. It will give relative deviation ≤1/3 allowable total error, conforming to the deviation range of conventional laboratories, which allows the calibration curve drawn by the new calibration solution to be used for the quantification of patient samples.
3. The calibration solution that passed the comparison should be packed and frozen as required, and relevant labels should be made to avoid repeated freezing and thawing for later usage. However, the following precautions should be taken when performing calibration solution lot replacement comparison in this way: (a). Even if the comparison succeeded, the theoretical value of each concentration of the new standard solution cannot be adjusted, and the theoretical value will be used in the subsequent volume; if the comparison failed, this lot of calibration solution should be discarded instead of using with adjusted calibration concertation value. (b). If the lot of calibration solution is updated quickly, it should be compared with the previous lot of calibration solution every time the comparison is performed. In the long run, it is necessary to pay attention to the continuous slope and relative deviation changes, so as to avoid systematic deviation in the process of multiple replacements. (c). It is also necessary to monitor long‐term stability and find suitable storage conditions, etc.

### 3.3 Application of reference materials as quality control materials in clinical mass spectrometry

In order to ensure the stable and reliable performance of the measurement method, the quality control methods that the laboratory can use include inter‐laboratory comparison and intra‐laboratory quality control methods, etc.

At this stage, because only a small number of laboratories is carrying out clinical mass spectrometry testing, each project has its own focus, and the quality of testing is uneven, it is necessary to combine the results for analysis when performing inter‐laboratory comparison, such as whether the grouping and target values are appropriate, whether the matrix of the comparison sample is similar to the actual test sample, whether the measurement method is consistent, the influence of the reference interval, etc.

At the same time, every project needs to adopt indoor quality control, and pay special attention to these following situations when implementing in accordance with industry standards: (1). The quality control substance should be uniform, stable and have similar or identical substrates, and its concentration should have a clinically significant concentration range; (2). when used for long‐term testing, quality control monitoring should be carried out in the form of Westgard, L‐J chat or a personalized quality control plan, instead of using the form of standard recovery rate; (3). During the implementation of the quality control, it should be recognized that: quality control out of control itself is not a big problem, but it is a huge problem if there is no loss of control or the judgment and treatment after the quality control if out of control and restoration of the of the state of control. This process can greatly help the laboratory to improve quality.

On the basis of the above, the author's laboratory also takes into account of other indictors of mass spectrometry technology, such as (1) whether the peak area response of the same lot of internal standard is stable; (2) whether the retention time of the analyte is stable; (3) calibration curve: *R* ^2^ > 0.990, the accuracy of SD1 at the lowest concentration point of the calibration curve should be 80%–20%, the other concentration points should be 85%–115%,^18^ and the linear slope and intercept should be kept stable every day.

### 3.4 Suggestions

At this stage, reference materials are receiving more and more attention from all parties, but the variety and quantity in the clinical field are still not enough to meet the demand. Clinical mass spectrometry technology as a new starting technology, due to the flexibility of its method and application in the LDT field, there is an increasing demand for reference materials, especially certified reference materials. Under this situation, it is necessary to increase the research input of reference materials and efficiency at the national level. However, this is a very slow process; at the same time, authoritative institutions, like the Clinical Laboratory Center of the National Health Commission, are also increasing the accuracy verification plans for urgently needed times year by year; the government or authoritative institutions could regularly evaluate and update the brand and article number of the reference materials used for calibration and quality control on their official websites to unify as much as possible the reference materials used by various laboratories national wide.

Reference materials play a key role in the accuracy and comparability of test results. In the absence of commercial kits and mature quality standards for mass spectrometry, how to ensure the accuracy and comparability of results within the laboratory and between laboratories is a problem that laboratories, authorities and government must face and strive to solve. National institutions, such as the Chinese Academy of Metrology or the Clinical Laboratory Center of the National Health Commission, can guide various laboratories to gradually unify calibrators by providing unified calibrator information; the laboratories can execute the relevant information issued by the authoritative organization, do a good job of confirming the measurement method at the beginning of the method, select the appropriate calibrator and carefully prepare and evaluate it, and carry out quality control in accordance with the standard, etc. Carefully understand and correctly regulate the use and management of reference materials to ensure the accuracy of test results, thereby improving the comparability and consistency of results between laboratories.

## CONFLICTS OF INTEREST

The authors declared that they have no competing interests.

## Data Availability

Data sharing is not applicable to this article as no new data were created or analyzed in this study.

## References

[1] Chengbin W . Clinical laboratory application of mass spectrometry. Chin J Lab Med. 2019;42(6):395‐398. doi:10.3760/cma.j.issn.1009-8158.2019.06.001

[2] Liqiao H , Qiaoxuan Z , Xianzhang H . Current clinical situation and challenge for liquid chromatography‐tandem mass spectrometry in quantitative detection. Chin J Lab Med. 2020;43(10):945‐950. doi:10.3760/cma.j.cn114452-20200619-00564

[3] Rongrong S , Guoping S , Xun S , et al. Traceability in clinical testing. Lab Med Clin. 2014;z2:144‐146. doi:10.3969/j.issn.1672-9455.2014.26.079

[4] Liu F . How to use clinical test reference materials correctly. Elect J Clin Med Literat. 2019;6(54):123.

[5] Fu Y , Gao X , Huang J . Quality Evaluation of in vitro diagnostic reagents and traceability of reference materials. J Mol Diagn Ther. 2017;9(2):73‐77, 87. doi:10.3969/j.issn.1674-6929.2017.02.001

[6] Wenxiang C , Ziyu S , Shu W , et al. Measurement traceability in laboratory medicine[J]. Chin J Lab Med. 2006;29(1):17‐19. doi:10.3760/j:issn:1009-9158.2006.01.007

[7] Yuan L , Liu Q , Dan Z . Discussion on traceability system establishment for physical and chemical testing in medicine. Zhongguo Ceshi. 2009;35(4):23‐25.

[8] Li M , Sun H , Hu B , et al. Observation of the correctness and consistency of the test results of the two prealbumin measurement systems traceable to the same standard substance. Linchuang Jianyan, 2017;35(9):704‐705. doi:10.13602/j.cnki.jcls.2017.09.16

[9] Shinan W . Evaluation of bias in the measurement results of cholesterol two calibrators and problems in application of calibrators. Zhongguo Wei Jian Za. 2010;20(3):602‐603.

[10] Weiyan Z , Chuanbao Z , Wenjun MA , et al. Standardization of aldosterone and harmonization of renin in China. Chin J Lab Med. 2020;43(3):245‐249. doi:10.3760/cma.j.issn.1009-9158.2020.03.010

[11] Chinese Society of Laboratory Medicine . The recommendation for management of laboratory developed tests in clinical laboratory. Chin J Lab Med. 2017;40(3):162‐164. doi:10.3760/cma.j.issn.1009-9158.2017.03.005

[12] Chinese Society of Laboratory Medicine . Recommendations for clinical application of liquid chromatography‐mass spectrometry. Chin J Lab Med. 2017;40(10):770‐779. doi:10.3760/cma.j.issn.1009-9158.2017.10.009

[13] Clinical Mass Spectrometry Committee, Chinese Medical Doctor Association of Laboratory Medicine . Consensus of method development and validation of liquid chromatography‐tandem mass spectrometry in clinical laboratories. Laboratory Medicine, 2019;34(3):189‐196.

[14] Yang Z , Huang J , Yu T , et al. Analysis of the current situation and countermeasures of the national standard materials of in vitro diagnostic reagents in my country. Chinese Journal of. Biologicals. 2015;07:765‐771.

[15] Quan Can XU , Bei LH , et al. Research on metrology characteristics of medical reference laboratory. Int J Lab Med. 2015;17:2610‐2613. doi:10.3969/j.issn.1673-4130.2015.17.076

[16] Rui Z , Jinming LI . How to use standard materials correctly in clinical laboratory. Chin J Lab Med. 2009;32(1):10‐14. doi:10.3760/cma.j.issn.1009-9158.2009.01.003

[17] Shengxia M , Zhun G . Brief introduction of administration of enterprise’s reference product in in‐vitro diagnostic reagent quality system. China Med Device Inform. 2011;17(10):46‐53. doi:10.3969/j.issn.1006-6586.2011.10.010

[18] Kaijuan W , Yuanyuan P , Lijun S . Discussion on guidelines for validation of analytical methods based on mass spectrometry. Chin J Lab Med. 2020;43(12):1166‐1171. doi:10.3760/cma.j.cn114452-20200717-00616

